# A Multi‐foci Sparse‐Aperture Metalens

**DOI:** 10.1002/advs.202309648

**Published:** 2024-03-14

**Authors:** Borui Xu, Wei Wei, Ping Tang, Jingzhu Shao, Xiangyu Zhao, Bo Chen, Shengxiang Dong, Chongzhao Wu

**Affiliations:** ^1^ Center for Biophotonics Institute of Medical Robotics School of Biomedical Engineering Shanghai Jiao Tong University Shanghai 200240 China

**Keywords:** multi‐foci metalenses, optical sparse aperture, polarization‐independent metalenses, two‐photon polymerization

## Abstract

Multi‐foci lenses are essential components for optical communications, virtual reality display and microscopy, yet the bulkiness of conventional counterparts has significantly hindered their widespread applications. Benefiting from the unprecedented capability of metasurfaces in light modulation, metalenses are able to provide multi‐foci functionality with a more compact footprint. However, achieving imaging quality comparable to that of corresponding single‐foci metalenses at each focal point poses a challenge for existing multi‐foci metalenses. Here, a polarization‐independent all‐dielectric multi‐foci metalens is proposed and experimentally demonstrated by spatially integrating single‐foci optical sparse‐aperture sub‐metalenses. Such design enables the metalens to generate multiple focal points, while maintaining the ability to capture target information comparable to that of a single‐foci metalens. The proposed multi‐foci metalens is composed of square‐nanohole units array fabricated by two‐photon polymerization. The focusing characteristic and imaging capability are demonstrated upon the illumination of an unpolarized light beam. This work finds a novel route toward multi‐foci metalenses and may open a new avenue for dealing with the trade‐off between multi‐foci functionality and high‐quality imaging performance.

## Graphical abstract

The polarization‐independent multi‐foci sparse‐aperture (MSA) metalens is able to generate multiple focal points conveniently, while maintaining the remarkable ability to capture target information and demonstrating high‐quality focusing characteristics and imaging capability. Such MSA metalens finds a novel route toward multi‐foci metalenses and serves as a promising tool for diverse and multiple tasks of imaging.

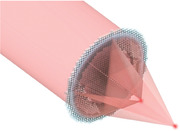

## 1 Introduction

Metalenses, composed of a monolayer artificial sub‐wavelength cells, have revolutionized the field of optics with their unprecedented capabilities to precisely manipulate incident electromagnetic waves by introducing local and abrupt phase shift.^[^ ^1^, ^2^ ^]^ This exceptional control allows for wavefront shaping on a wavelength scale, thus opening up new possibilities for developing ultrathin devices that can be seamlessly integrated into compact platforms. Metalenses are widely recognized as essential optical components, showing remarkable potential across various fields such as generation of light beam with specific wavefront,^[^ ^3^, ^4^ ^]^ imaging^[^ ^5^, ^6^ ^]^ and sensing.^[^ ^7^, ^8^ ^]^ Ultrathin design and straightforward manufacturing process make metalenses highly desirable for integrated optics and compact optical systems. Furthermore, metalenses provide functionalities that conventional lenses struggle to achieve, expanding the capabilities of existing optical systems in practical applications. Consequently, researchers have developed various types of metalenses, including broadband achromatic metalenses,^[^ ^5^, ^6^, ^9^, ^10^, ^11^, ^12^, ^13^, ^14^ ^]^ varifocal metalenses,^[^ ^15^, ^16^, ^17^ ^]^ and multi‐foci metalenses.^[^ ^18^, ^19^, ^20^, ^21^ ^]^

Among these advancements, multi‐foci metalenses attract special attention as they enable a single incident beam to focus at different positions along either the longitudinal or transverse direction. This unique functionality has been widely used in virtual reality displays,^[^ ^22^ ^]^ microscopic imaging,^[^ ^23^ ^]^ particle manipulation,^[^ ^24^ ^]^ holography,^[^ ^25^ ^]^ optical communications,^[^ ^26^ ^]^ and optical spectroscopy.^[^ ^27^ ^]^ One popular manner to achieve multi‐foci capability is to incorporate parts of individual single‐foci metalenses concentrically into a larger metalens.^[^ ^18^, ^28^ ^]^ There are significant challenges in such manner to achieve the desired multi‐foci effect due to cross‐talk among the unit cells of metalenses with different focal points, the consequent sacrifice of spatial sampling resolution and the effective aperture of each individual sub‐metalens. Additionally, another type of multi‐foci metalenses has demonstrated the ability to exhibit polarization‐dependent characteristics based on the Pancharatnam‐Berry (PB) phase or plasmonic nanorod, facilitating the focusing of *x*‐/*y*‐polarized or left/right circularly polarized light to distinct positions, which limits the available number of focal points.^[^ ^19^, ^20^, ^21^ ^]^ Hence, there is a compelling need for the development of an innovative methodology for multi‐foci metalenses that can efficiently capture target information at a reduced cost, while maintaining polarization‐independent characteristics. Thus far, optical sparse‐aperture metalenses have demonstrated their effectiveness in reducing the size requirements of a fully‐filled aperture metalens by integrating multiple smaller sub‐apertures.^[^ ^29^, ^30^, ^31^ ^]^ A sparse aperture combines signals from multiple sub‐apertures to produce an image with a resolution that matches that of an aperture equivalent to the circumcircle encompassing all sub‐apertures. Inspired by this concept, the area saved by employing a sparse‐aperture design can be populated by other sparse‐aperture metalenses, each focusing on distinct points, thereby achieving a multi‐foci metalens at a relatively low expense of target information. Due to the significant attenuation caused by the interaction between electromagnetic waves and free electrons in the metal, the transmission efficiency of the metallic metalenses in the visible spectrum is remarkably diminished,^[^ ^19^, ^32^ ^]^ severely limiting its practical implementation. Alternatively, dielectric nanostructures with materials such as silicon (Si), titanium oxide (TiO_2_), gallium nitride (GaN) and IP‐Dip photoresist have shown prospect in overcoming the weakness of metallic nanostructures.^[^ ^2^, ^33^, ^34^ ^]^ Considering the limitation of polarization‐dependent metalens on light source and transmission efficiency, design of polarization‐independent nanostructures for the generation of multiple focal points is of great significance. Therefore, it is highly desirable to integrate the outstanding attributes of optical sparse‐aperture and dielectric nanostructures into multi‐foci metalenses, while ensuring polarization‐independent characteristics.

In this study, we introduce and experimentally demonstrate a polarization‐independent all‐dielectric multi‐foci sparse‐aperture (MSA) metalens. The proposed MSA metalens consists of multiple sub‐sectors. Crucially, this design is not a random assembly of sub‐sectors; instead, it represents a carefully engineered configuration, guided by the principles of optical sparse aperture. Each pair of diagonal sub‐sectors, termed as a sparse‐aperture sub‐metalens, is able to focus light to the same point, achieving diffraction‐limited performance at each focal point. Each sub‐sector is composed of polarization‐independent IP‐Dip square‐nanohole units array fabricated by two‐photon polymerization, suitable for focusing and imaging at a wavelength of 650 nm. To the best of our knowledge, it is the first time to design and demonstrate a multi‐foci metalens enabled by optical sparse aperture, offering high‐quality and conveniently‐generated multiple focal points, which holds promise for advancing the development of multi‐foci optical elements in virtual reality displays, microscopic imaging and various applicable scenarios.

## 2 Design of All‐Dielectric MSA Metalens

**Figure 1:**
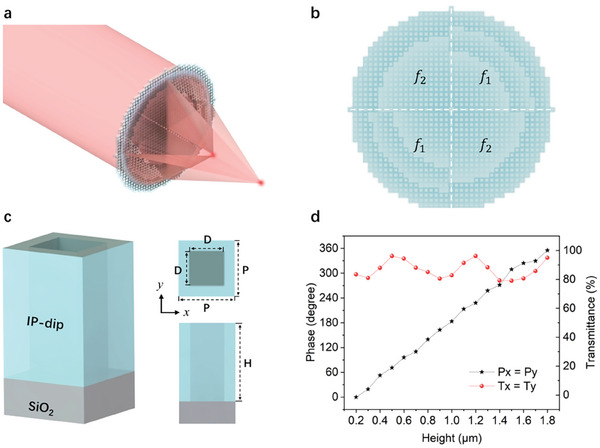
Design and principle of the multi‐foci sparse‐aperture (MSA) metalens. a) Artistic schematic of the light focused by the all‐dielectric MSA metalens. b) Schematic of the all‐dielectric MSA metalens with two focal points, *f_1_* = 0.5 mm and *f_2_* = 1.0 mm, respectively. c) Schematic of square‐nanohole unit cell with 3D view, top view and front view, where *D*, *P*, *H* refers to the length of hollow edge, period of unit cells and height of nanohole, respectively. d) Simulated results: plots of phase and transmittance shift of the transmitted light through the square‐nanohole unit cells with different heights ranging from 0.2 to 1.8 µm under 650 nm normal incident light, where *Px*, *Py*, *Tx*, and *Ty* refer to the relative phase and transmittance shift under *x*‐polarized incident light and *y*‐polarized incident light, respectively.

**Figure** **1**a schematically shows the MSA metalens composed of quadrantally‐distributed sub‐sectors with two focal points at a working wavelength of 650 nm. The MSA metalenses with three and four focal points have also been designed (see Section S1, Supporting Information, for details). Each sub‐sector consists of periodically arranged square‐nanohole units array made of IP‐Dip photoresist. Each pair of diagonal sectors forms an independent sparse‐aperture sub‐metalens with an effective aperture diameter of 100.5 µm, capable of focusing light at designated focal point while causing light to diverge at other positions, effectively minimizing the interference between each other. Thus, the MSA metalens composed of sparse‐aperture sub‐metalenses can efficiently focus light into two focal points: *f_1_* = 0.5 mm and *f_2_* = 1.0 mm, as depicted in Figure 1b. In order to achieve the desired focusing effect, the spatial and spectral phase profiles of each sparse‐aperture sub‐metalens are determined using the following equation:^[^ ^35^ ^]^

(1) $$\varphi_{i}\;\left(x,y\right)=\;2n\pi -\frac{2\pi }{\lambda }\left(\sqrt{{f_{i}}^{2}+x^{2}+y^{2}}-f_{i}\right)$$

where (*x, y*) correspond to the spatial coordinates, *φ_i_* (*x, y*) represents the corresponding spatial phase profile, *n* represents an arbitrary integer, *f_i_* denotes the focal length of the *i*th sparse‐aperture sub‐metalens, and the wavelength of the incident light is denoted by *λ* = 650 nm. To realize the desired phase profiles, IP‐Dip square‐nanohole units array were directly designed and fabricated on a SiO₂ substrate. Figure 1c illustrates the configuration of square‐nanohole unit cell, with the period (*P*) set as 500 nm and the hollow edge length (*D*) set as 300 nm. Simulation results demonstrate that square‐nanohole unit cells with varying heights (*H*) ranging from 0.2 to 1.8 µm can cover the transmission phase modulation ranging from 0 to 2π, and maintain high transmittance (>79%) under both *x*‐polarized and *y*‐polarized normal incident light, as depicted in Figure 1d. Due to fundamental principle of polarized light decomposition, the transmission phase and magnitude of square‐nanohole unit cell can remain identical regardless of the incident light's polarization. The utilization of square‐nanohole unit cells as waveguides allows for the achievement of phase modulation, based on propagation phase function:^[^ ^36^, ^37^, ^38^ ^]^

(2) $$\varphi \;\left(x,y,H\right)=\frac{2\pi }{\lambda }\;n_{\mathrm{eff}}\left(x,y,\lambda \right)H$$

where *n*_eff_ represents the effective refractive index and *H* denotes the height of square‐nanohole unit cells.

## 3 Simulation of All‐Dielectric MSA Metalens

**Figure 2:**
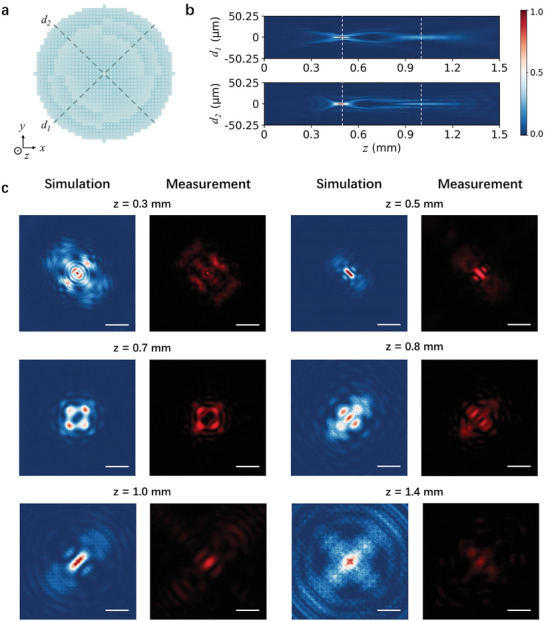
Simulated and measured focusing characteristics of the MSA metalens. a) Schematic representation of the all‐dielectric metalens with well‐defined *x*, *y*, *z*, *d_1_* and *d_2_* axes. b) Simulated normalized intensity distribution of the transmitted light in *d_1_‐z* plane and *d_2_‐z* plane. c) Simulated and measured normalized intensity distribution along *z* direction of the transmitted light in *x‐y* plane when *z* = 0.3, 0.5, 0.7, 0.8, 1.0, and 1.4 mm, respectively. Scale bar is 20 µm.

Numerical simulations were conducted to analyze the focusing performance of the designed all‐dielectric MSA metalens, as shown in **Figure** **2**, where the *x*, *y*, *z* axes, and the two orthogonal axes *d_1_* and *d_2_* are indicated in Figure 2a. The *d_1_* and *d_2_* axes align with the diagonal direction of the MSA metalens (represented by the two dark dashed lines). Figure 2b illustrates the normalized intensity distribution of transmitted light in *d_1_‐z* plane and *d_2_‐z* plane for the MSA metalens with two designed focal points located at *f_1_* = 0.5 mm and *f_2_* = 1.0 mm. Starting from *z* = 0 mm, the light gradually converges toward *z* = 0.5 mm (indicated by the left white dashed lines), and then diverges until refocusing at *z* = 1.0 mm (indicated by the right white dashed lines). To gain further insight into this phenomenon, the light intensity distribution in *x‐y* plane at specific positions: *z* = 0.3, 0.5, 0.7, 0.8, 1.0, and 1.4 mm were calculated and measured, as shown in Figure 2c. A plane wave source at 650 nm was used as the light source. The simulation results clearly demonstrate that light spots away from the focal length exhibit a divergent characteristic. For a more comprehensive evaluation, complete light intensity distribution along *z* direction has also been provided (see Section S3, Supporting Information, for details). The simulation results reveal that light spots at *z* = 0.5 and 1.0 mm correspond to the minimum full width at half maximum (FWHM) in their adjacent range, indicating the well‐defined focusing characteristics. As the light source is plane wave source, the images in the focal planes at *z* = 0.5 and 1.0 mm can be used as the point spread functions (PSFs) of the MSA metalens. Based on the same principle, the MSA metalenses with three and four focal points were also designed and characterized numerically (see Section S1, Supporting Information, for details).

**Figure 3:**
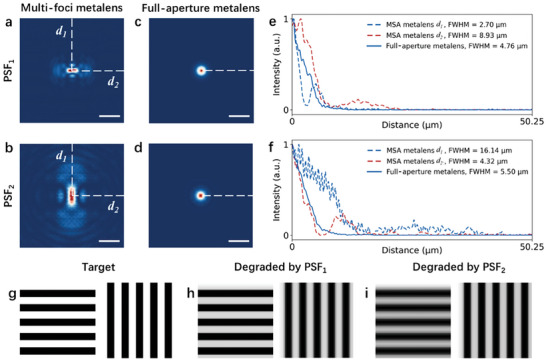
Simulated imaging performance of the MSA metalens. a,b) The point spread functions (PSFs) of the MSA metalens at the focal lengths *f_1_* = 0.5 mm and *f_2_* = 1.0 mm, respectively. c,d) The PSFs of corresponding single‐foci full‐aperture metalenses. Scale bar is 20 µm. e,f) A comparison of the 1‐D PSFs along the *d_1_* axis and *d_2_* axis of the MSA metalens and the corresponding single‐foci full‐aperture metalenses. g–i) Target objects consisting of horizontal and vertical bars and the generated images obtained by convolving the target objects with the PSFs of the MSA metalens.

In the case of a conventional full‐aperture metalens and single‐foci sparse‐aperture metalens, the image can be generated by convolving the target object with the PSF of the metalens:^[^ ^29^ ^]^

(3) $$g\;\left(x,y\right)=\;f\left(x,y\right)*\mathrm{PSF}\left(x,y\right)+n\left(x,y\right)$$

where * represents the convolution operation, and *f*(*x, y*) and *g*(*x, y*) correspond to the system input (target object) and system output (acquired image), respectively. PSF(*x, y*) refers to the point spread function (PSF) of the imaging system, while *n*(*x, y*) represents the additional noise acquired during the imaging process. Since the MSA metalens is composed of two sparse‐aperture sub‐metalenses, the same approach in Equation 3 is also applicable to calculate the generated image. The PSF_1_ of the MSA metalens, corresponding to the intensity distribution at *z* = 0.5 mm, has a FWHM of 2.70 µm along the *d_1_* direction and 8.93 µm along the d_2_ direction as shown in **Figure** **3**a,e. In contrast, the PSF_2_, corresponding to the intensity distribution at *z* = 1.0 mm, has a FWHM of 16.14 µm along the *d_1_* direction and 4.32 µm along the *d_2_* direction as shown in Figure 3b,f. The FWHMs of PSF_1_ and PSF_2_ for the respective single‐foci full‐aperture metalens are calculated to be 4.76 and 5.50 µm, as shown in Figure 3c–f. Although the MSA metalens demonstrates reduced FWHM values in its PSFs, the inclusion of sidelobes inevitably leads to a degradation in resolution for the MSA metalens. Furthermore, the uneven intensity distribution of the PSF profiles along different direction is the inherent characteristics of sparse‐aperture metalenses,^[^ ^29^, ^30^ ^]^ resulting in anisotropic imaging resolution, as shown in Figure 3g–i. Specifically, for images generated from convolving target objects with PSF_1_, the MSA metalens exhibits high resolution for horizontal bars shown with sharper edges, demonstrating its superior ability in vertical orientation. In contrast, it displays lower resolution for vertical bars, suggesting a decreased capability to resolve details in horizontal orientation. Nevertheless, for images generated from convolving target objects with PSF_2_, the imaging resolution along horizontal direction is higher than that along vertical direction. In order to address the issue of uneven distributed PSFs, a MSA metalens with consistent FWHM value in all directions was also designed (see Section S2, Supporting Information, for details). It consists of 6 subsectors that form two sub‐metalenses. As illustrated in Figures S3 (Supporting Information), the incident light converges to the intended focal points at *z* = 0.5 and 1.0 mm as designed, while diverging at other positions. The FWHM value of PSFs at *f_1_* = 0.5 mm and *f_2_* = 1.0 mm are 3.85 and 7.96 µm, respectively.

## 4 Fabrication and Characterization of All‐dielectric MSA Metalens

### 4.1 Fabrication of MSA Metalens

**Figure 4:**
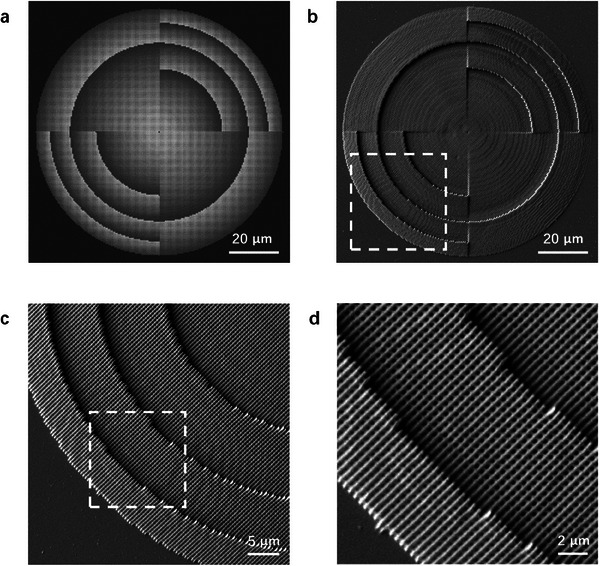
Model and scanning electron microscopic (SEM) images of the fabricated MSA metalens. a) Model image of the all‐dielectric MSA metalens employed for the two‐photon polymerization process. The grayscale value of each pixel in the image is proportional to the actual height of corresponding position of the metalens. b–d) SEM images of the fabricated MSA metalens with different magnifications. The region enclosed by the white dashed box corresponds to the area of the image captured at a higher magnification.

The fabricated MSA metalens was then characterized using a scanning electron microscope (SEM) as shown in **Figure** **4**. The SEM images illustrate the skeleton of the fabricated metalens with clear and intact nanoholes, consistent with the designed model.

### 4.2 Optical Characterization of MSA Metalens

**Figure 5:**
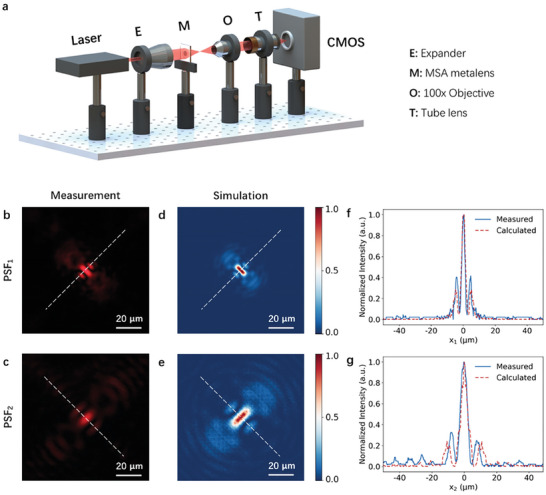
a) Schematic of optical focusing experimental setup for the fabricated MSA metalens. b–e) Comparison of the measured and simulated 2‐D PSFs. f‐g) Comparison of the measured and simulated 1‐D PSFs along the diagonal cut represented by the white dashed lines in b‐e.

The focusing capability of the all‐dielectric MSA metalenses was characterized through an optical setup, illustrated in **Figure** **5**a. Beam profiles along *z* direction of the all‐dielectric MSA metalens are presented in Figure 2c, which are highly consistent with the simulation results. A comprehensive characterization necessitates the consideration of the complete light intensity distributions at different locations along *z* direction, thus corresponding results have also been provided (See Section S3, Supporting Information, for details). As shown in Figure S5 (Supporting Information), starting from *z* = 0 mm, the light progressively converges up until *z* = 0.5 mm, then diverges until refocusing at *z* = 1.0 mm. The performance differences between the simulation and measurement can be effectively demonstrated by examining the diagonal cuts of the PSFs, as shown in Figure 5b‐g. It can be found that a close correlation between both sets of data is obvious. The presence of a relatively higher intensity side peak in the measured PSF_1_ and higher level of noise in the PSF_2_ can be attributed to the fabrication defects and inevitable measurement error. The simulation results indicated that our MSA metalens can achieve a focusing efficiency of up to 65.76% at *f_1_* and 41.71% at *f_2_*, respectively. And the experimental counterparts, as measured by the focus spot, are 52.15% at *f_1_* and 29.98% at *f_2_*, respectively, which are relatively lower than the simulation results. This difference may be attributed to the discrepancy in size of the square nanohole as well as defects in the fabricated MSA metalens.

**Figure 6:**
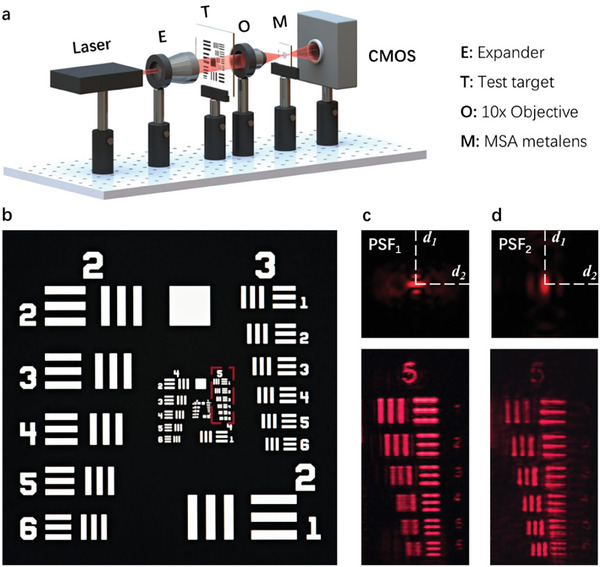
Imaging with the fabricated MSA metalens. a–b) Schematic illustration of the imaging setup and a negative 1951 United States Air Force (USAF) target. The Group 5 area enclosed by the red dashed line represents the target imaging region. c–d) Captured image of the USAF test target using the MSA metalens at *λ* = 650 nm.

The imaging capability of the MSA metalens was further validated using a negative 1951 United States Air Force (USAF) target, as depicted in **Figure** **6**a,b. The resolution chart comprises horizontal bars, vertical bars and number patterns with the horizontal bars orientated along the *d_1_* direction, and the vertical bars along the *d_2_* direction. The corresponding images were captured with the CMOS camera, successfully rendering the resolution test target with a maximum resolution of 12.4 µm, corresponding to the space distance of line pairs in element 3 of Group 5. Initially, a clear image of the horizontal bars was obtained, with the vertical bars appearing blurred, as shown in Figure 6c. Upon moving the CMOS camera away from the metalens, the image clarity was reversed: vertical bars were clearly visible, while horizontal bars appeared blurred, as shown in Figure 6d. The two images correspond to the results produced by PSF₁ and PSF₂, respectively, consistent with the simulation results, as shown in Figure 3.

## 5 Conclusion

In summary, this work presents the design, fabrication and characterization of a novel polarization‐independent all‐dielectric MSA metalens. This metalens consists of four quadrantally distributed sub‐sectors forming two sparse‐aperture sub‐metalenses, featuring two distinct focal points along the propagation direction. The experimental intensity distribution of each focal point matched the theoretical predictions, demonstrating an excellent agreement between theory and experiment. The imaging experiment shows the remarkable capability of the MSA metalens, achieving a maximum imaging resolution of 12.4 µm. MSA metalenses with three and four focal points has also been demonstrated, indicating its flexibility and high degree of design freedom. More importantly, both the focal length and the number of focal points can be customized to meet specific requirements, thus opening up potential applications in various fields such as virtual reality display, microscopic imaging, holography and optical spectroscopy.

## 6 Experimental Section

### Finite‐Difference Time Domain (FDTD) Simulations

FDTD simulations were conducted using Lumerical's FDTD solver. In the simulation of phase and transmittance shift, a unit cell on a fused silica substrate was simulated with periodic boundary conditions for *x* and *y*, and perfectly matched layers on top and bottom. A plane wave source was placed inside the substrate and below the unit cell, and a frequency‐domain field and power monitor was placed above the unit cell to collect transmitted power and phase. The refractive index of IP‐Dip is set to 1.548 at 650 nm.

### Physical Optics Propagation (POP) Method

POP simulations were conducted using Zemax OpticStudio. In the simulation of light intensity distribution in *x‐y* plane along *z* direction, initially, FDTD simulation of a whole MSA metalens was conducted to obtain the near‐field transmitted power and phase. The near‐field results were then exported as a *zbf* file and imported into Zemax OpticStudio to calculate the far‐field light intensity distribution.

### Angular Spectrum Method

To calculate the normalized intensity distribution of the transmitted light in *d_1_‐z* plane and *d_2_‐z* plane, the angular spectrum method was utilized based on the phase profile determined by Equation 1 and transmittance results of square‐nanohole unit cells.^[^ ^39^ ^]^

### Two‐Photon Polymerization

MSA metalens was fabricated on a 0.7 mm thick fused silica substrate using “dip‐in” two‐photon polymerization. In order to enhance the stability of the metalens on the substrate, a buffer layer with a thickness of 200 nm was introduced between the substrate and the metalens structure. The utilization of two‐photon polymerization allows to exploit the phenomenon of two‐photon cross‐linking within the focal volume of an ultrafast laser pulse, enabling the creation of intricate 3D structures in a single step, as stated in previous reports.^[^ ^40^, ^41^ ^]^ For the fabrication process, the Nanoscribe Photonic Professional GT2 system is employed in conjunction with IP‐Dip photoresist (Nanoscribe GmbH, Germany) and a 63x objective lens in dip‐in mode. The shape feature of each unit cell was represented by a 5×5 PNG image with grayscale value directly proportional to the actual height. The whole PNG image of the designed metalens with 1005×1005 pixels, as shown in Figure 4a, was then imported into the processing package as printing template. The hatching and slicing distance for the MSA metalens were set to 50 nm using continuous heightfield approach. The stage velocity was maintained at 200 µm s^−1^. Due to the 200 nm minimum voxel line width achievable through two‐photon polymerization using a 63x objective lens, the nanohole structures were written as multiple lines using the Galvo scan mode and piezo z‐axis writing mode. The optimal laser power was determined to be 40 mW, to avoid undesirable bubble in the structures, and maintain the completeness of the fabricated structures. The sample underwent a 40 min development process using PGMEA (Aladdin Chemical Reagent Co., Ltd) to eliminate any unpolymerized resin remnants. Subsequently, the sample was immersed directly in isopropyl alcohol (China National Pharmaceutical Group Corporation) for 5 min to remove PGMEA.

### Optical Characterization

A supercontinuum laser (SuperK EVO) was used for illumination. In focusing test, the unpolarized 650 nm incident beam emitted from supercontinuum laser was expanded and collimated via a beam expander (JMLASER) and then focused by the MSA metalens. The beam profiles along *z* direction were magnified by a combination of a 100x objective lens and a tube lens, prior to being captured by a complementary metal‐oxide‐semiconductor (CMOS) camera. In imaging, an expanded and collimated 650 nm beam passed the resolution test chart and was then focused by an objective lens. This lens ensured that the diverging light was tightly focused on the MSA metalens, covering a broad area of imaging target.^[^ ^13^ ^]^

## Conflict of Interest

A patent application is related to this work (Number 202410267316.0). The authors declare that they have no other conflicts of interest.

## Supporting information

Supporting Information

## Data Availability

The data that support the findings of this study are available from the corresponding author upon reasonable request.
