# Supplementary material for: A Multi‐foci Sparse‐Aperture Metalens
Source: Adv Sci (Weinh). 2024 Mar 14;11(19):2309648. doi: 10.1002/advs.202309648 (PMC11109648; doi:10.1002/advs.202309648)
Supplement: Supplementary file 1 — Supporting Information [file ADVS-11-2309648-s001.pdf]

ADVANCED SCIENCE
Open Access

Supporting Information

for *Adv. Sci.*, DOI 10.1002/advs.202309648

A Multi-foci Sparse-Aperture Metalens

*Borui Xu, Wei Wei, Ping Tang, Jingzhu Shao, Xiangyu Zhao, Bo Chen, Shengxiang Dong and Chongzhao Wu**

A Multi-foci Sparse-Aperture Metalens

Borui Xu, Wei Wei, Ping Tang, Jingzhu Shao, Xiangyu Zhao, Bo Chen, Shengxiang Dong and Chongzhao Wu
Center for Biophotonics, Institute of Medical Robotics, School of Biomedical Engineering, Shanghai Jiao Tong University, Shanghai, China

S1. Simulation of the all-dielectric MSA metalens with three and four focal points.

The MSA metalens with three and four focal points was demonstrated numerically. As illustrated in Figure S1 and S2, the incident light converges to the intended focal points as designed, while diverging at other positions.

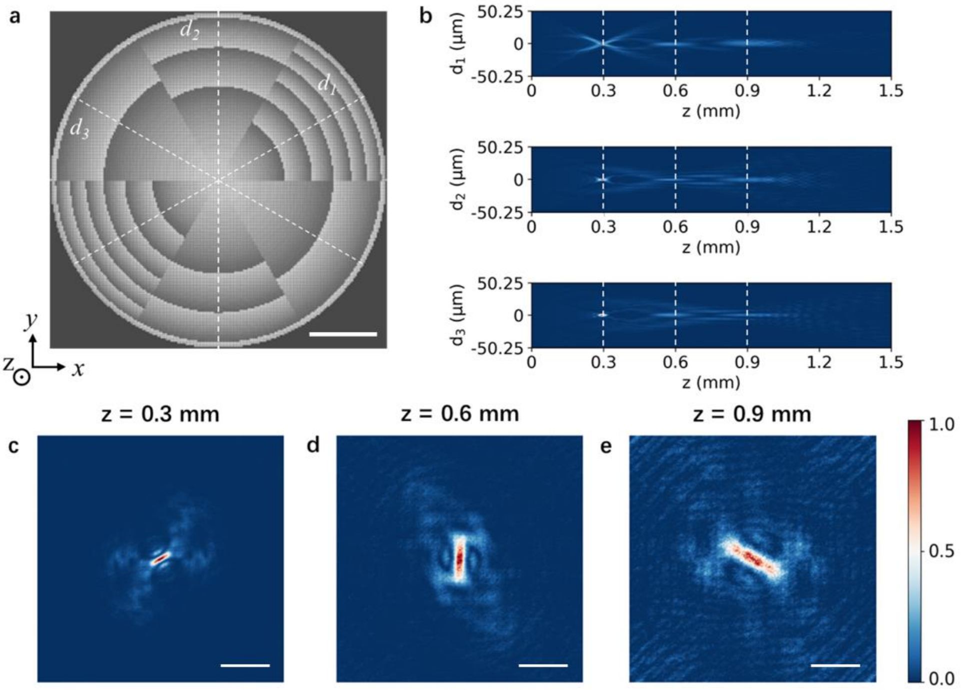

Figure S1. Focusing characteristics of the MSA metalens with three focal points f_1 = 0.3 mm, f_2 = 0.6 mm and f_3 = 0.9 mm. a) Schematic representation of the metalens with well-defined x, y, z, d_1, d_2 and d_3 axes. b) Simulation results of the normalized intensity distribution of the transmitted light in d_1-z, d_2-z and d_3-z planes. c-e) Simulation results of the normalized intensity distribution of the transmitted light in x-y plane when z = 0.3 mm, 0.6 mm and 0.9 mm. Scale bar is 20 µm.

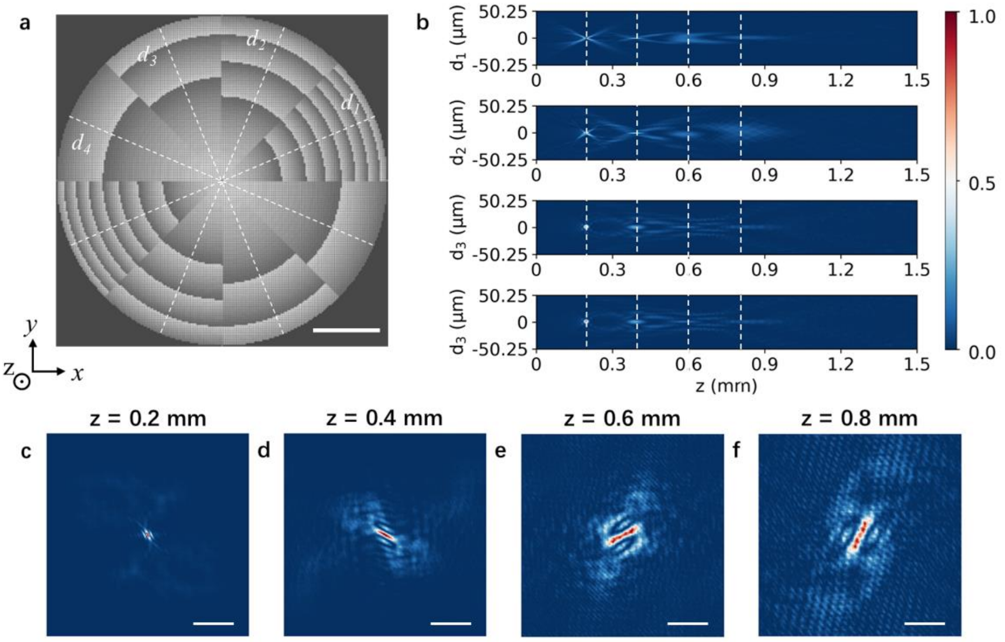

Figure S2. Focusing characteristics of the MSA metalens with four focal points f_1 = 0.2 mm, f_2 = 0.4 mm, f_3 = 0.6 mm and f_4 = 0.8 mm. a) Schematic representation of the metalens with well-defined x, y, z, d_1, d_2, d_3 and d_4 axes. b) Simulation results of the normalized intensity distribution of the transmitted light in d_1-z, d_2-z, d_3-z and d_4-z planes. c-f) Simulation results of the normalized intensity distribution of the transmitted light in x-y plane when z = 0.2 mm, 0.4 mm, 0.6 mm and 0.8 mm. Scale bar is 20 µm.

S2. Simulation of the all-dielectric MSA metalens with consistent FWHM value in all directions.

The MSA metalens with consistent FWHM value in all directions was demonstrated in Figure S3. It consists of 6 subsectors that form two sub-metalenses and the incident light converges to the intended focal points at z = 0.5 mm and 1.0 mm as designed, while diverging at other positions. The FWHM value along x-axis of PSFs at f_1 = 0.5 mm and f_2 = 1.0 mm are 4.27 µm and 7.76 µm, respectively. The FWHM values along y-axis of PSFs at f_1 = 0.5 mm and f_2 = 1.0 mm are 3.85 µm and 7.96 µm, respectively.

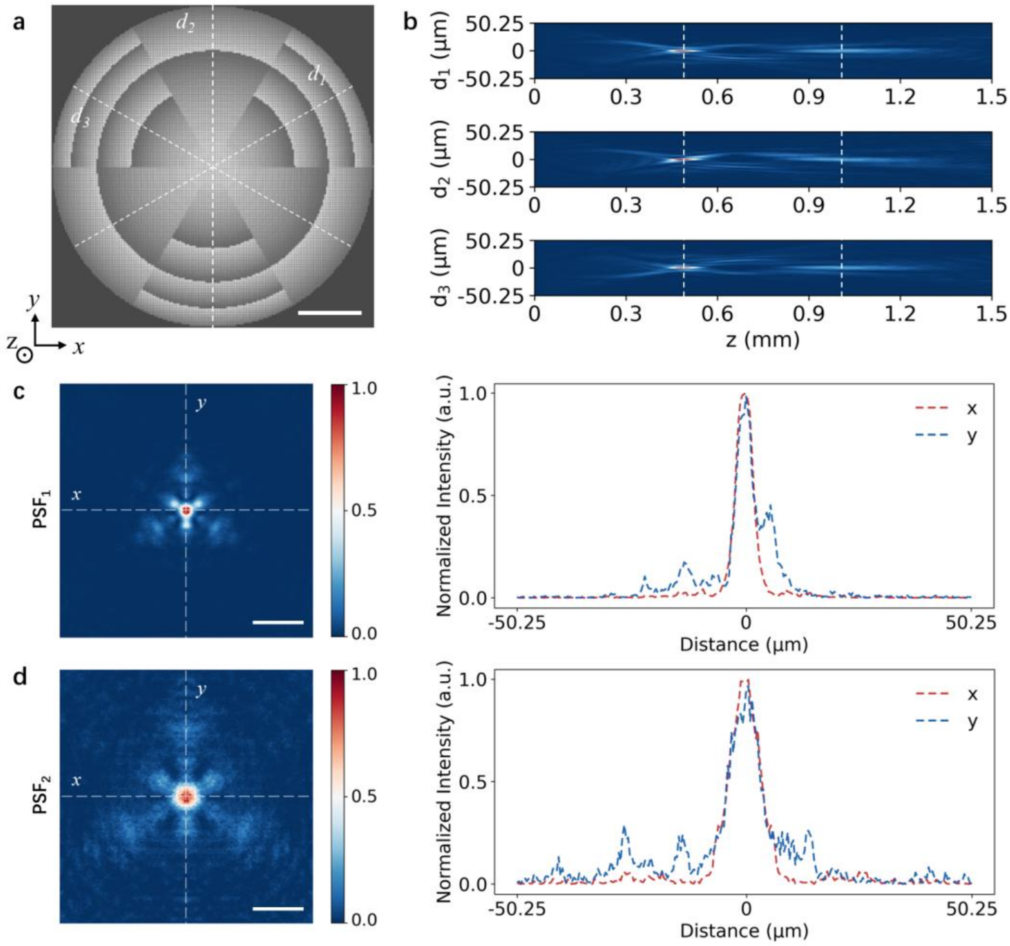

Figure S3. Focusing characteristics of the MSA metalens with consistent FWHM value in all directions. a) Schematic representation of the metalens with well-defined x, y, z, d_1, d_2 and d_3 axes. b) Simulation results of the normalized intensity distribution of the transmitted light in d_1-z, d_2-z and d_3-z planes. Normalized light intensity distribution along x and y axes (represented by the white dashed lines) of the PSFs at c) f_1 = 0.5 mm and d) f_2 = 1.0 mm, respectively. Scale bar is 20 µm.

S3. Light intensity distribution in *x-y* plane along *z* direction.

To conduct a more detailed investigation into the focusing performance of lenses and

comprehensively compare simulation and experiment results, the simulated and measured light intensity distribution in *x-y* plane along *z* direction are shown in Figure S4 and Figure S5. Despite some differences mainly attributed to fabrication issues, the measurement results are generally consistent with the simulation results.

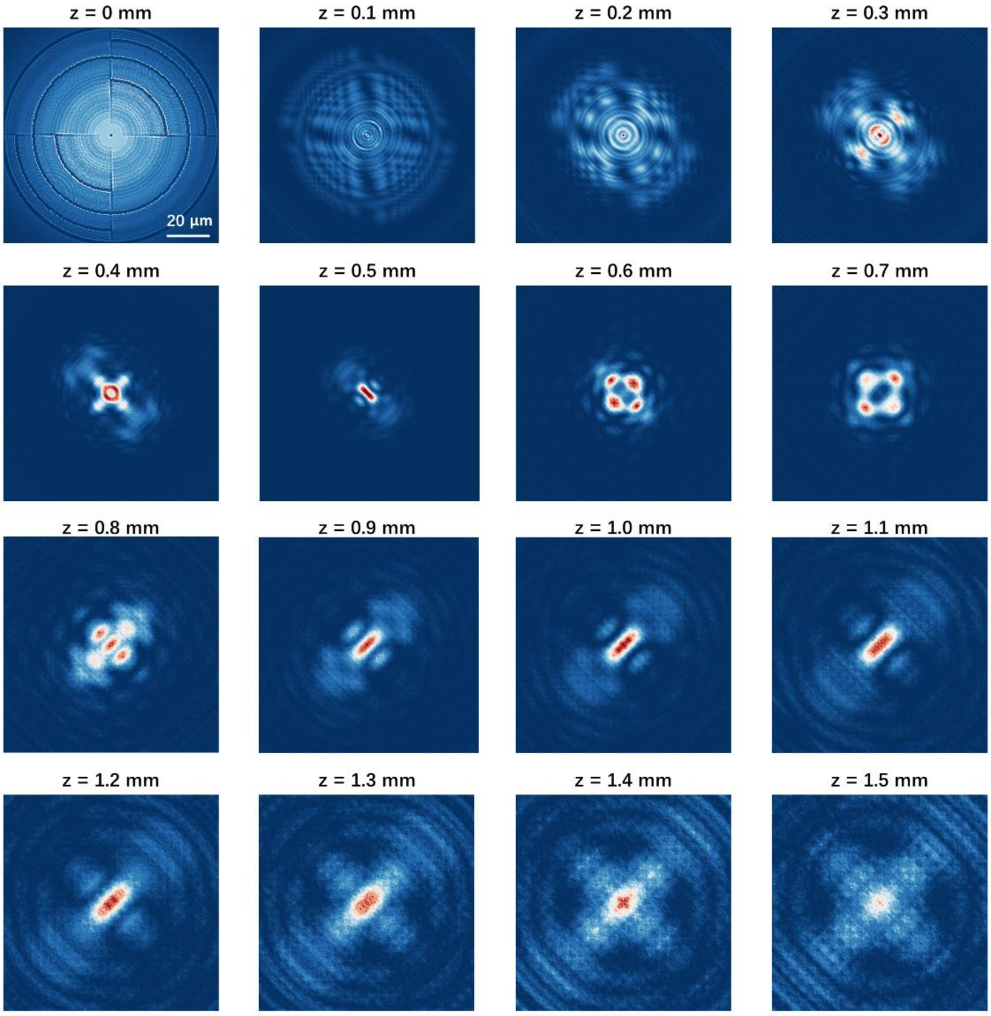

Figure S4. Simulation results of normalized electrical intensity distribution in *x-y* plane along *z* direction.

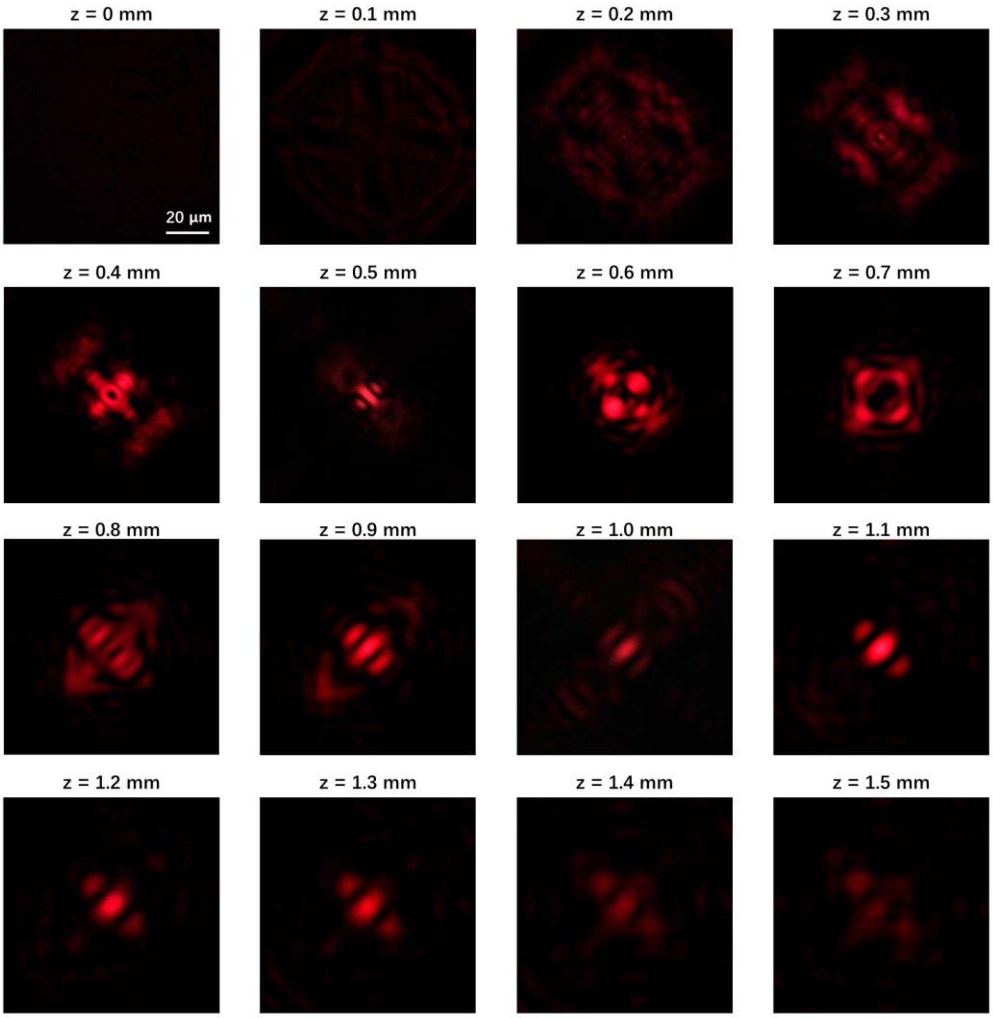

Figure S5. Measurement results of normalized electrical intensity distribution in *x*-*y* plane along *z* direction.

S4. Optical properties of two-photon polymerized IP-Dip at visible wavelengths.

The transmittance of two-photon polymerized IP-Dip is >95% at the wavelength of 633 nm and IP-Dip is essentially transparent up to the wavelength of 1.4 µm.[1] The real part of the refractive index of the two-photon polymerized IP-Dip at visible wavelengths is shown in Figure S6.

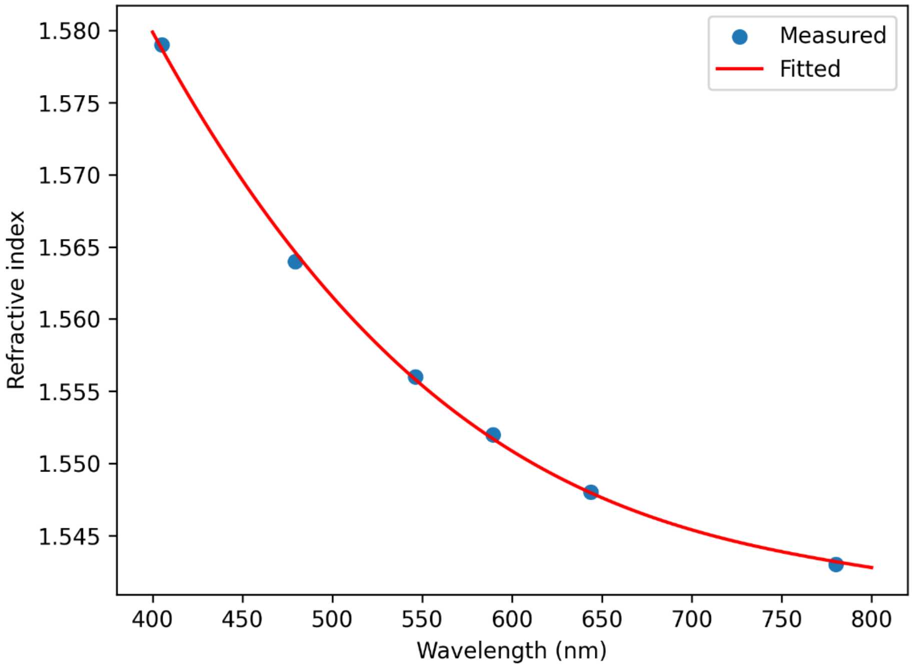

Figure S6. Measured and third-order polynomial-fitted refractive index of two-photon polymerized IP-Dip in the visible light wavelength range.

S5. MTF comparison of the MSA metalens and the corresponding full-aperture metalens.

The modulation transfer function (MTF) is derived from the PSF of an optical system by taking the two-dimensional Fourier transform of the PSF. This process converts the spatial domain information of the PSF into its frequency domain representation. The calculation of MTF can be expressed as follows:

$$\mathrm{MTF}(f_x, f_y) = \left| \frac{\mathcal{F}\{\mathrm{PSF}(x,y)\}}{\iint \mathrm{PSF}(x,y)\, dxdy} \right| \tag{S1}$$

where $\mathcal{F}$ denotes the Fourier transform, PSF(x, y) is the point spread function in the spatial domain, and $|\cdot|$ represents the magnitude of the complex Fourier transform. The MTF(f_x, f_y) is a function of spatial frequency (f_x, f_y), providing a measure of ability in an optical system to preserve the contrast of spatial details at various frequencies. For the MSA metalens, the MTF_1 along vertical axis and MTF_2 along horizontal axis initially exhibit a sharp decrease in the middle frequency range, followed by an increase,

and the cut-off frequency closely approaches that of the full-aperture metalens, as shown in Figure S7. However, the MTF_1 along horizontal axis and MTF_2 along vertical axis for the MSA metalens remain significantly lower than that of the full-aperture metalens in the entire frequency range. In summary, the MSA metalens is able to maintain imaging capabilities comparable to the full-aperture metalens in one axis while significantly lagging behind in the other direction.

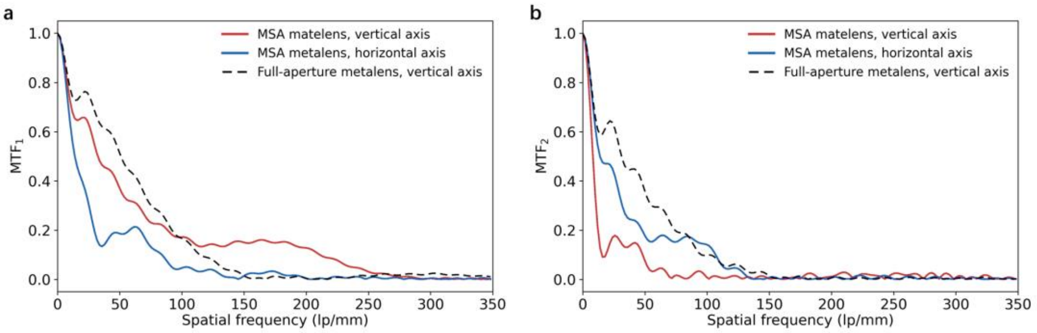

Figure S7. 1-D MTF comparison of the MSA metalens and corresponding full-aperture metalens at a) f_1 = 0.5 mm and b) f_2 = 1.0 mm, respectively.

S6. Deconvolution by a Wiener filter.

Wiener filter is based on the least squares method, which is extensively employed for the purpose of image restoration. The foundational principle of this filter is to diminish the mean square error (MSE) that exists between the reconstructed image $f'(x, y)$ and the original image $f(x, y)$, striving for the minimum possible value of this error.

$$e^2 = E\left[\left(f(x,y) - f'(x,y)\right)^2\right] \tag{S2}$$

The Wiener filter operates as an algorithm for image restoration within the frequency domain. The calculation of the spectral components of the restored image is conducted according to the following expression:

$$\mathrm{H}(f_x, f_y) = \frac{\mathcal{F}\{\mathrm{PSF}(x,y)\}}{\iint \mathrm{PSF}(x,y)\,dxdy} \tag{S3}$$

$$F'(f_x, f_y) = \frac{H^*(f_x, f_y)G(f_x, f_y)}{\left|H(f_x, f_y)\right|^2 + C} \tag{S4}$$

where $H(f_x, f_y)$ signifies the optical transfer function (OTF) and $G(f_x, f_y)$ denotes the Fourier transform of the degraded image $g(x, y)$. Moreover, $H^*(f_x, f_y)$ represents the complex conjugate of $H(f_x, f_y)$, and the C value is indicative of the ratio between the power spectrum of the noise and that of the original image. For the experimental phase, C is adjusted to an optimized value derived from the outcomes of the restoration process. The sharp edges of the pattern captured by the MSA metalens are restored by a Wiener filter, as shown in Figure S8.

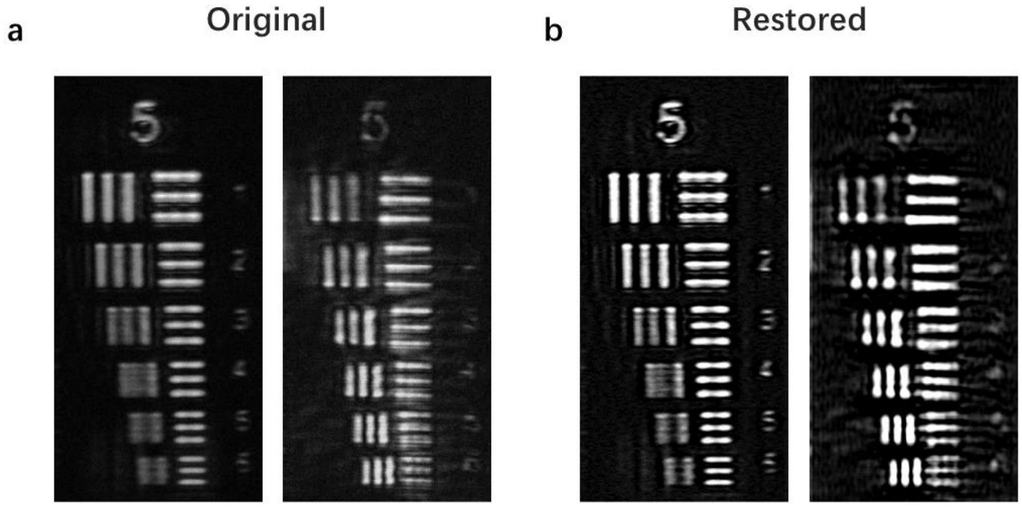

Figure S8. a) Original grayscale images captured by the MSA metalens and b) restored images by a wiener filter.

S7. Electric field distributions of the square nanohole unit cells.

The waveguide resonance can be excited inside the square nanohole unit cells, which can be directly observed from the simulated electric field distributions, as shown in Figure S9. The induced electric fields are highly concentrated inside the nanohole.

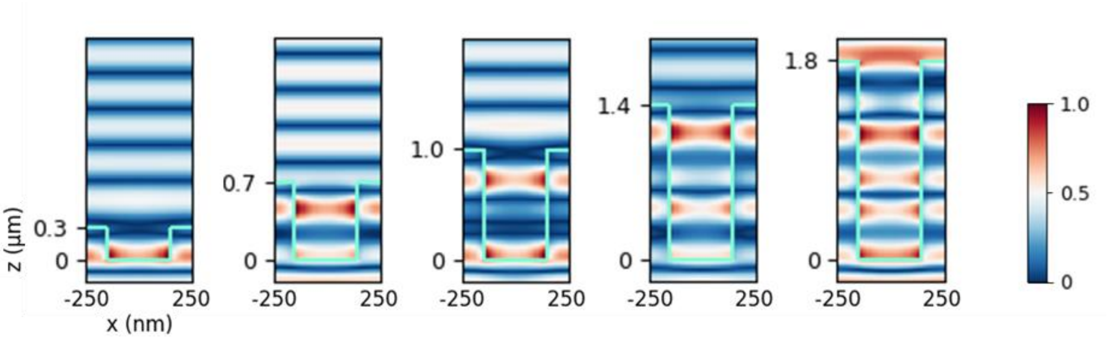

Figure S9. Simulation results of the normalized electric field distributions inside the square nanohole unit cells with period (P) = 500 nm, hollow edge length (D) = 300 nm and various height (H) of 0.3μm, 0.7μm, 1.0μm, 1.4μm, and 1.8μm, respectively. The light blue lines highlight the boundaries of the square-nanohole and z refers to the direction of height.

References

[1] Y. Li, S. Park, M. McLamb, M. Lata, S. Schöche, D. Childers, I. D. Aggarwal, M. K. Poutous, G. Boreman, T. Hofmann, *Opt. Mater. Express* **2019**, 9, 4318.
